# Short-term clinical and radiological outcome of the uncemented isoelastic monoblock Affinis Glenoid vitamys in stemless anatomic total shoulder arthroplasty: a multicenter study

**DOI:** 10.1016/j.jseint.2026.101687

**Published:** 2026-03-05

**Authors:** Agnieszka Halm-Pozniak, Philip Kasten, Max J. Kääb, Marco Greis, Sebastian Gros, Alexander Berth

**Affiliations:** a Department of Orthopaedic Surgery, Otto-von-Guericke-University, Magdeburg, Germany; b Orthopädisch Chirurgisches Centrum (OCC), Tübingen, Germany; c Sporthopaedicum Straubing, Straubing, Germany; d University Hospital Regensburg, Clinic for Trauma Surgery, Regensburg, Germany; e MVZ Marienstift Arnstadt, Arnstadt, Germany; f Helios Park-Klinikum Leipzig, Leipzig, Germany

**Keywords:** Uncemented glenoid, Isoelasticity, Anatomic shoulder arthroplasty, Radiological outcome, Clinical outcome, Survival, Shoulder, Osteoarthritis

## Abstract

**Background:**

This study evaluated the short-term clinical and radiological outcomes of an uncemented isoelastic monoblock glenoid component in stemless anatomic total shoulder arthroplasty (aTSA). We hypothesized that the implant's design and biomechanical properties would offer sufficient fixation and good functional outcomes.

**Methods:**

In this prospective multicenter study, 75 patients received stemless aTSA for shoulder osteoarthritis using the Affinis Glenoid vitamys implant. We evaluated 64 patients at a mean follow-up of 25.2 ± 2.1 months. The Constant Score, Simple Shoulder Test, active range of motion, and complications were recorded. The radiological evaluation was based on the occurrence of radiolucent lines (RLLs) and periprosthetic bone adaptations using plain radiographs and scored according to Lazarus and Molé.

**Results:**

Clinical scores and range of motion outcomes improved significantly after surgery (*P* < .001). The radiological evaluation revealed minor humeral periprosthetic bone adaptations without clinical consequences. RLLs on the glenoid side were detected in 31.5% of cases; most (62%) of them were < 2 mm. The mean adapted Molé score was 1.08 ± 2.34. Since the majority of observed RLLs were not located around the pegs, 95% of the investigated implants were classified 0 according to Lazarus. Finally, no revision surgery was necessary due to failed fixation or loosening of the glenoid component.

**Conclusion:**

Stemless aTSA with the uncemented isoelastic monoblock Affinis Glenoid vitamys yielded good and reliable short-term radiological and clinical results. Studies with a longer follow-up period are necessary to confirm these results.

Numerous studies have proven that the clinical use of anatomic total shoulder arthroplasty (aTSA) with a conventional cemented polyethylene (PE)-glenoid is a safe and effective treatment option for glenohumeral osteoarthritis.^24^^,^^39^^,^^40^^,^^44^^,^^45^ However, the increased risk for a revision as a result of aseptic glenoid loosening and/or rotator cuff (RC) insufficiency remains a serious concern regarding the long-term survival of aTSA with a cemented PE glenoid.^2^^,^^13^^,^^43^ Therefore, aTSA is currently the subject of controversial debate, particularly in elderly patients, as a revision to a reverse total shoulder arthroplasty (TSA) may be necessary due to a secondary RC failure.^45^ In younger patients, however, the most common reason for revisions in aTSA is a mechanical failure of the glenoid component by loosening, PE wear, displacement, and/or implant breakage.^50^^,^^52^ Over the last 2 decades, numerous innovations in prosthetic design, surgical techniques, and implant materials aimed to increase the survivorship of anatomic glenoid components.^35^^,^^44^^,^^53^ The modern concepts include modularity and convertibility to a reverse TSA, new fixation strategies (eg hybrid fixation, use of trabecular metal) to secure primary fixation and subsequently improve secondary implant stability as well as the possibility to address bone loss, and deformities by augmented glenoid components.^1^^,^^25^^,^^36^ These advancements have shown promising early results with lower occurrence of radiolucent lines (RLLs), as well as loosening and revision rate compared to the early glenoid components.^16^^,^^17^^,^^19^

A new concept is the use of isoelastic materials. This study evaluated the clinical and radiological results of aTSA using an uncemented, monoblock glenoid component, which has an elastic modulus similar to bone. The biomechanical characteristics of this implant were successfully used in total hip arthroplasty for many years.^22^^,^^48^^,^^12^ We hypothesized that this glenoid component could provide sufficient primary implant stability and successful osseointegration. Therefore, the radiological analysis of X-rays focused on the detection of RLLs and further potential radiographic changes around the glenoid component and their clinical impact.

## Materials and methods

### Patients

This prospective, multicenter, noncomparative study enrolled 75 patients from 5 centers in Germany treated with primary stemless aTSA using an uncemented isoelastic monoblock glenoid between January 2020 and June 2021 by primary or secondary osteoarthritis of the shoulder. The inclusion and exclusion criteria are shown in detail in Table I. The study was performed in accordance with the standards of the 1964 Declaration of Helsinki and was approved by the Institutional Review Board of the Medical School, Otto-von-Guericke University of Magdeburg (148/17). Written informed consent was obtained from all patients prior to inclusion in the study.

**Table I:** Inclusion and exclusion criteria.

Inclusion criteria
Primary OA of the shoulder stage III or IV according to Kellgren and Lawrence^31^
Post-traumatic OA of the shoulder/humerus fracture sequelae type 1a according to the modified Boileau classification by Kimmeyer et al^7^^,^^37^
Secondary OA of the shoulder by synovial chondromatosis
Dislocation arthropathy stage 2 or 3 according to Buscayret^9^
Persistent pain at rest with loss of shoulder function despite conservative treatment for more than 12 mo
Intact rotator cuff confirmed by preoperative MRI
Cross sectional imaging (MRI): glenoid retroversion <15°, humeral head subluxation <70%
Exclusion criteria
Prior shoulder surgery to the affected shoulder
Known or suspected noncompliance (eg drug or alcohol abuse)
Rheumatoid arthritis
Neuromuscular or other skeletal disorders
Metabolic bone diseases and bone interacting medical-treatment

*OA*, osteoarthritis; *MRI*, magnetic resonance imaging.

### Surgical technique and implants

All surgeries were performed by senior shoulder surgeons with the patients in a beach chair position via a standard deltopectoral approach under general anesthesia in combination with an interscalenic block.

**Figure 1:**
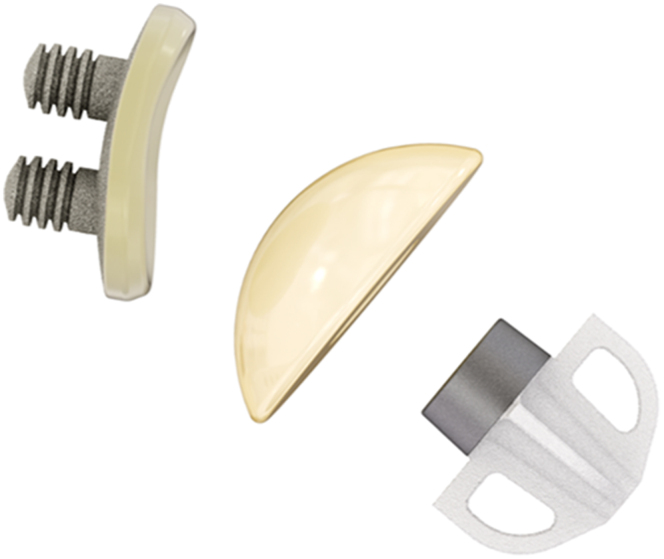
The Affinis Short stemless prosthesis system with the Affinis Glenoid vitamys uncemented glenoid component (Mathys Ltd. Bettlach/Enovis).

The patients were treated with the Affinis Short stemless shoulder arthroplasty system (Mathys Ltd. Bettlach/Enovis) (Fig. 1). The stemless humeral component was a metaphyseal fixation device made from titanium with 4 fins, each containing a window for bone ingrowth. The components were additionally coated with calcium phosphate. The surgeons selected the stemless prosthesis based on the preoperative subject assessment of bone quality (eg, bone density, presence of cysts) on radiographs and magnetic resonance imaging (MRI) scans as well as the patient's activity level, age, and comorbidities. Therefore, none of them were intended to be treated with a stemmed prosthesis.

The glenoid replacement was performed with the Affinis Glenoid vitamys, which is an uncemented monoblock component made of vitamin E-enriched, highly cross-linked polyethylene (HXLPE). It has an elastic modulus similar to bone. The backside and the pegs of the implant were coated with a thin layer of titanium particles (Fig. 1).

After exposure of the proximal humerus and removal of osteophytes, the humeral head was resected using the manufacturer's resection device. Subsequently, the cancellous bone of the metaphysis was prepared according to the manufacturer's instructions. For glenoid preparation, a circumferential release of the labrum and capsule was performed to achieve an adequate glenoid exposure. Then, a guided K-wire was placed centrally to ensure accurate positioning of the implant with regard to orientation and inclination, and for precise subsequent preparation of the glenoid. Through the K-wire, a stepwise glenoid reaming was performed to the planned size. Eccentric reaming was performed by B-type glenoids. Additional bone or osteophytes were removed if needed. Two peg holes were then drilled using a K-wired guide and the glenoid trial was inserted to confirm the correct sizing. Then, the glenoid implant was placed with an impactor. Accurate bone-implant alignment was assured. The surgeons did not use augmented glenoid components in this study. Finally, the metaphyseal fixation device was impacted almost entirely in the prepared cancellous humeral bone. The ceramic humeral head was fixed through a Morse taper by hand and the whole prosthesis was then ultimately positioned until the head is sitting flush on the resection plane.

### Clinical evaluation

Primary clinical and functional outcomes included the active range of motion (ROM) measured by using a goniometer in the scapular plane (forward elevation, extension), in the neutral position of the arm at the side (external rotation), the absolute and age- and gender-adjusted Constant-Murley-Score (CS),^10^ and the Simple Shoulder Test (SST).^21^ Preoperative evaluations were performed 1 day prior surgery. The postoperative follow-up (FU) visit was at a minimum of 24 months postoperatively (Table II). Complications were also noted. All measurements were performed by the same person, who was one of the authors and/or surgeons.

**Table II:** Baseline characteristics.

Characteristic	Value
Number of shoulders, n	75
Age at surgery, yr, mean ± SD (range)	66 ± 10 (34-84)
Sex n (%)	
Male	42 (56)
Female	33 (44)
Operated side, n (%)	
Right	38 (51)
Left	37 (49)
Follow-up, months, mean ± SD (range)	25.2 ± 2.1 (24.1-30.4)
Glenoid morphology, n (%)	
A1	37 (49)
A2	22 (29)
B1	10 (13)
B2	6 (8)
Indication for surgery, n	
Primary OA of the shoulder	68
Posttraumatic OA of the shoulder/Humerus fracture sequelae	4
Secondary OA by synovial chondromatosis	1
Dislocation arthropathy	2
Glenoid size distribution, n (%)	
1	20 (28)
2	40 (53)
3	11 (15)
4	4 (5)

*SD*, standard deviation; *OA*, osteoarthritis.

### Radiographic evaluation

Radiographic evaluation was carried out using anteroposterior and axillary views. All radiographs were taken by a certified radiologist and medical radiologic technologists, all of them experienced and specialized in musculoskeletal radiology. Special care was taken to assess the positioning of the scapula and humeral rotation when analyzing changes in component position. The RC integrity was assessed preoperatively in the MRI. The glenoid morphology was classified according to Walch et al^51^ and MRI images suitable for basic bone visualization were included in these analyses if the preoperative axial radiographs were of limited use for a proper assessment.^6^^,^^34^

The glenoid version angle was calculated using the method of Friedman^18^ and the evaluation of the posterior humeral head subluxation was carried out taking according to Walch and Domos.^14^^,^^51^

The radiographs were analyzed regarding the presence of RLLs or other periprosthetic bone adaptions (bone resorption, stress shielding). The radiographic assessment was performed separately by 5 authors (A.H.-P., P.K., M. K., M. G., S. G., A. B.), all of them experienced orthopedic surgeons. If RLLs were present, the width was measured in millimeters using CHILI digital radiology software system (CHILI Web software, V4.0).

**Figure 2:**
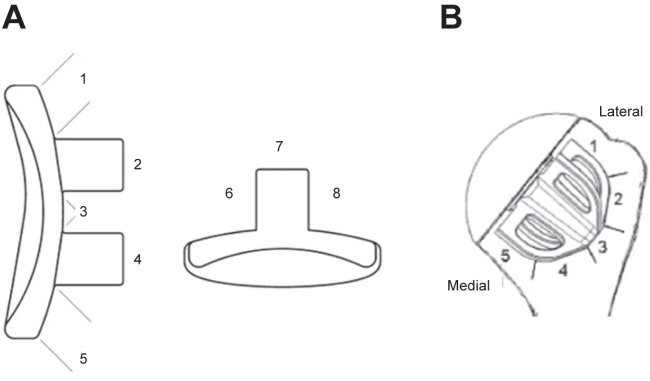
Regions of interest of the glenoid in true ap and axillary view (**A**) and humerus component in true ap view (**B**).

The RLLs around the pegged glenoid component were graded by 8 zones and measured for width according to Greiner et al (Fig. 2)^23^ and scored according to Lazarus and Molé.^41^ For radiographic analysis of the stemless humeral component, 5 regions of interest were defined around the implant according to Bell et al (Fig. 2).^4^

The original Molé score only evaluates RLLs on anteroposterior X-rays of keeled glenoids. Thus, we used a modified Molé score, which was adapted to be used for a two-pegged design and added measurements of RLLs in axillary radiographs.^23^

### Statistical analysis

We performed all statistical analyses with the Statistical Analysis System (version 9.4; SAS Institute Inc., Cary, NC, USA).

Unless otherwise specified, results are given as mean ± standard deviation. A *P* value of <0.05 was considered statistically significant. A sample size of 50 was considered to reach 95% statistical power to detect a difference between the null hypotheses with a significance level (alpha) of 0.05 using a 2-sided 1-sample *t*-test. The Wilcoxon signed rank test was used to compare the preoperative to postoperative values of the functional outcome parameters. The correlations between the RLLs versus clinical outcome and versus glenoid type were calculated with the Fisher exact test. The intraclass correlation coefficient (ICC) was calculated for assessment of interobserver reliability using measurements on 30 randomly selected postoperative radiographs by 5 authors.

### Postoperative protocol

Postoperatively, patients were instructed to wear a neutral rotation sling for six weeks. The early postoperative rehabilitation program was standardized across all centers and included passive, non–weight-bearing upper extremity exercises over the initial 6 weeks: supported pendulum exercises and isometric deltoid muscle activation as well as gentle active mobilization of the elbow, wrist, and hand. In the supine position, patients were allowed active external rotation up to 20° and forward elevation up to 90°. After six weeks, active external rotation progressively increased, and full forward elevation within pain-free limits and shoulder internal rotation gradually increased to full ROM, excluding heavy lifting. Muscle-strengthening exercises and sports activities were allowed over the following three months.

## Results

### Patients

The data of the 75 subjects enrolled in the present study are summarized in Table II. A total of 10 patients were lost to FU, 3 patients died unrelated to the surgery, and 7 were unable to visit the hospital due to personal reasons during the pandemic, leaving 65 patients that were evaluated. In this cohort, 1 radiographic evaluation was not feasible due to poor image quality. Data were excluded from the evaluation, although the humerus and glenoid implants were not considered loose.

### Clinical assessment

The preoperative and postoperative functional status (CS, SST, and active ROM) of the patients is presented in Table III. The functional assessment showed a significant postoperative improvement compared to preoperative results for all outcome parameters (*P* < .001).

**Table III:** Preoperative and postoperative values of Constant Score, Simple Shoulder Test, and active range of motion.

Parameter	Preoperative	24 mo postoperative	*P* value
Absolute CS (points)	27.2 ± 12.2	70 9 ± 14.4	<.001
Adjusted CS (points)	36.0 ± 15.1	96.3 ± 20.9	<.001
SST (points)	2.4 ± 2.4	10.1 ± 2.1	<.001
Forward elevation (°)	86 ± 72	152 ± 24	<.001
Extension (°)	20 ± 8.1	25 ± 11.6	<.001
External rotation (°)	21 ± 14	51 ± 18	<.001

*CS*, Constant-Murley Score; *SST*, Simple Shoulder Test.

Data given as means ± standard deviations.

### Complications

There were 2 (3%) complications during the study period. One complication occurred intraoperatively during the preparation of the glenoid component. During the reaming process, the reamer was damaged. A 1-size smaller reamer was therefore used and accordingly the patient was treated with a smaller glenoid component. Another patient suffered from acute shoulder pain and loss of function on the affected side four weeks after surgery that was caused by a forced abduction and external rotation during the outpatient rehabilitation program. The clinical suspicion of a subscapularis tendon lesion could not be confirmed by ultrasound or MRI, so we suspected that an overstretching/overloading of the subscapularis tendon was responsible for the complaints. The subsequent conservative treatment was successful, pain symptoms diminished after two weeks, and radiographs during FU demonstrated proper alignment and centering of the humeral component.

### Radiographic assessment

The radiological evaluation during the FU was available for 64 (85%) of the 75 shoulders. The interobserver reliability of the evaluation of the postoperative radiographs was substantial for the glenoid component (ICC 0.661; 95% confidence interval 0.442 to 0.805) and almost perfect for the humeral component (ICC 0.902; 95% confidence interval 0.824 to 0.947). The preoperative evaluation of glenoid morphology (Walch-type, retroversion) and extent of posterior humeral head subluxation are shown in Table II. In general, the radiological evaluation of the humerus and glenoid components during the FU in both groups did not present a loosening of implant nor was any component classified “at risk” for loosening.

### Glenoid implant

RLLs around the glenoid implant were found in 31.5% of the cases (n = 20/64). Of these, 9 patients had RLLs in 1 zone, 5 patients in 2 zones, 3 patients in 3 zones, 2 patients in 4 zones, and 1 patient in 5 zones. The mean adapted Molé score^41^ was 1.08 ± 2.34 points (range, 0 to 11). The extent and distribution of the RLLs in relation to the evaluated ROI is shown in Table IV.

**Table IV:** Mean adapted RLLs score by glenoid zones.

Glenoid zone	n RLLs	<1.0 mm	1–2 mm	>2 mm	Mean RLLs score∗
(Points/zone)		(1 point)	(2 points)	(3 points)	(Max. 24 points)
1	5 (8%)	2 (3%)	2 (3%)	1 (2%)	0.14
2	1 (2%)	0 (%)	1 (2%)	0 (0%)	0.03
3	6 (9%)	3 (5%)	2 (3%)	1 (2%)	0.16
4	2 (3%)	1 (2%)	1 (2%)	0 (0%)	0.05
5	11 (17%)	7 (11%)	2 (3%)	2 (3%)	0.27
6	7 (11%)	3 (5%)	3 (5%)	1 (2%)	0.19
7	2 (3%)	1 (2%)	1 (2%)	0 (0%)	0.05
8	9 (14%)	5 (8%)	4 (6%)	0 (0%)	0.20
Mean	43 (67%)	22 (36%)	16 (26%)	5 (9%)	1.08 ± 2.34

*n*, number of cases; *RLL*, radiolucent lines.

∗ Adapted Molé score according to Greiner et al.

Since the majority of observed RLLs were not located around the pegs, 95% (n = 61/64) of the investigated components were classified 0 according to Lazarus.^38^

The RLLs were assessed in relationship to the glenoid type according to Walch.^51^ We found 60% of RLLs in type B2, 38% in type A2, and 30% in type B1 (Table V). However, no correlation was found between glenoid morphology and appearance of RLLs (*P* = .28).

**Table V:** Correlation between glenoid type according to Walch and the occurrence of RLLs.

Glenoid type	None	Any RLLs >0 mm	Total
A 1	22 (79%)	6 (21%)	28 (44%)
A 2	13 (62%)	8 (38%)	21 (77%)
B 1	7 (70%)	3 (30%)	10 (16%)
B 2	2 (40%)	3 (60%)	5 (8%)
Total	44 (69%)	20 (31%)	64 (100%)

*RLL*, radiolucent lines.

Furthermore, there were no statistical differences between the CS (*P =* .12) and SST (*P* = .25) and glenoid types according to Walch.^51^ Additionally, no correlation was found between the adapted Molé score^41^ and postoperative CS (Spearman correlation of 0.07, *P =* .60) and SST (Spearman correlation of 0.04, *P =* .75).

### Humerus implant

RLLs at the 2-year FU examination were detected in 1 (1.5%) case measuring ≤1 mm and in zone 5 around the stemless humeral component according to Bell et al.^5^ Comparing the anteroposterior radiographs at 2-year FU with the postoperative radiographs, a decrease of bone mineral density in the area of the greater tuberosity was observed in 2 (3.1%) cases. We also detected partial exposure of the fins underneath the humeral head component in 1 (1.5%) stemless humeral implant. Finally, a minimal bone resorption in the calcar region in the FU radiographs was found in 3 (4.6%) cases. All of these periprosthetic bone reactions are signs of minimal stress shielding with no influence on the functional outcome during the short-term FU.

## Discussion

**Figure 3:**
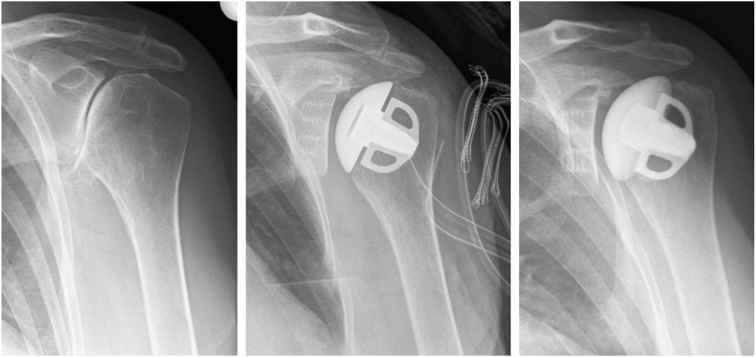
True ap radiographs of a 66-year-old female with primary osteoarthritis of the *left* shoulder undergoing aTSA with the Affinis Short stemless prosthesis and an Affinis Short Bionit ceramic head combined with an Affinis Glenoid vitamys uncemented glenoid component. Radiographs taken preoperatively, immediately postoperatively, and at 24 months postoperatively. *aTSA*, anatomic total shoulder arthroplasty.

This prospective multicenter study demonstrates excellent short-term clinical and radiological results of stemless aTSA at a minimum of two years FU using a novel uncemented isoelastic monoblock glenoid component (Fig. 3). In general, the findings in the present study showed significant improvements in pain assessment, functional scores, and ROM. The radiographic assessment showed only a low number of RLLs on the glenoid side, suggesting a sufficient implant stability.

The glenoid implant design investigated in this study is based on the idea of the isoelastic features of RM Pressfit vitamys cup model (Mathys Ltd., Bettlach, Switzerland; Bettlach/Enovis, Udine, Italy) successfully used in total hip arthroplasty, which has led to excellent long-term clinical and radiographic outcomes.^26^ It consists of HXLPE stabilized with vitamin E resulting in a higher resistance to oxidation, aging, and abrasion than conventional PE types.^3^ Interestingly, this HXLPE component shows elastic modulus equivalent to human cancellous bone.^3^ The potential biomechanical benefit of the “isoelasticity” allows a force transmission from the implant to the periprosthetic bone stock that is comparable to physiological conditions.^32^ This properties may diminish stress shielding, a potential risk for implant failures.^28^ The primary stability of the implant used in this study relies on pressfit fixation of the 2 central pegs in an appropriately prepared glenoid bone bed.^20^ However, in order to enable sufficient secondary and long-term stability, osseointegration of the implant into the surrounding bone tissue is crucial.^32^ To support this process, the surface with contact to the periprosthetic bone as well as the anchoring pegs of the implant are coated with a layer of pure titanium particles, leaving a coarsely porous surface structure to enhance the structural connection between the bone and the implant.^42^^,^^46^ Another advantage of this glenoid component and its surface is a nonmodular design. This “monoblock” design may be a promising solution for glenoid implant failure modes caused by creating several interfaces in modular shoulder components.^27^ Besides the beneficial aspects of “modularity,” various studies have shown that corrosion, fretting, PE wear or metal debris still remain major concerns in modular prosthesis.^11^^,^^15^

The main findings of this study are the radiographic outcomes where 69% of the shoulders had no RLLs around the glenoid at 24 months. Moreover, RLLs of >2 mm were rare and confined to the zones at the glenoid base (zones 1, 3, and 5) and not around the pegs (zones 2, 4, and 7). These radiological findings suggest a sufficient bony ingrowth of the 2 central pegs, which is essential prerequisite for the stability of uncemented pegged glenoid implants.^8^^,^^20^ However, the RLLs rate of 31% after 2 years reported in this study may arouse some concerns and requires critical analysis. First, the physiological variation of the glenoid version and inclination as well as the positioning of the patient during the X-ray may influence the projection of the glenoid component and also the assessment of RLLs. Despite the standardized internal protocol and experienced radiologists, some deviations of a few degrees from an ideal alignment cannot be ruled out. Second, design-related factors may also have influenced the appearance of RLLs in this study. It can be speculated that the thin, radiopaque titanium layer on the convex PE surface facing the reamed sclerotic bone could simulate RLLs. These typical appearance of the “double lines” with a gap <1 mm are located behind the baseplate on the edges of the glenoid component and can be detected directly on the postoperative X-rays. These immediate minor gaps show no progression during the FU and are distinct from those, that develop over time and are based on different mechanism (wear, loosening). A recent experimental study of Kasten et al^30^ support this assumption that the tilting of this novel, uncemented, titanium-coated PE glenoid in relation to the X-ray could affect the appearance of RLLs.

Compared with other contemporary cemented, pegged PE glenoid components investigated at similar or longer FU periods,^23^^,^^33^ we observed fewer and less pronounced RLLs. The possible explanations are the biomechanical properties, the monoblock PE component, and the isolelasticity of this new implant. Moreover, there was a significant difference in FU (5 years FU by Greiner vs. 2 years FU in our study) that may also influence the occurrence of the RLLs. In contrast, a similar amount of shoulders (71.2%), as reported in this study, remained free of glenoid RLLs in a recent study on aTSA using vitamin E infused all-PE cemented glenoid at a median FU of 35.3 months.^17^ However, the authors of this study observed glenoid osteolysis in 5.7% of cases,^17^ unlike our results where no osteolysis occurred. This may be related to the titanium coating of the implant used in the present study, which may have promoted osseointegration. The second possible explanation is the difference in the investigated PE components. The one used in our study was a double pegged high cross-linked PE, while the one evaluated by Entezari was a moderately cross-linked PE with peripheral peg. The fact that most of the RLL in this study were located at convex surface of the glenoid and not around the pegs explains the very low average Lazarus score. Additionally, the observed fraction of shoulders with a Lazarus grading of 0 (95%) was greater than that reported for cemented all-PE glenoid with a similar two-peg design at ≤ 5 years (73.3%).^23^ This finding was further supported by the mean Molé score achieved in this study, which was lower than that reported with cemented PE glenoids at ≤5 years (1.1 ± 2.3 versus 2.9 ± 3.4).^23^

On the other hand, some authors found no significant differences in the rate of glenoid RLL between uncemented and cemented PE glenoids at 6 years postoperatively.^22^ However, these comparisons must be interpreted with caution given the differences in PE types, overall design features, FU periods, and methodological differences in radiological evaluation potentially modifying radiographic findings.

The results of this study showed that the evaluated clinical scores and ROM values improved significantly after surgery, and the appearance of RLLs did not influence the postoperative clinical outcome during the FU. No radiographic signs of glenoid component failure or loosening were observed. However, it still must be evaluated in further studies what effect the radiographic changes around the glenoid component used in this study may have on midterm and long-term clinical outcomes or implant survival.

Moreover, in the present study, glenoid morphology did not have significant impact on the appearance of RLLs during the 2-year FU. In contrast, the study of Greiner et al^23^ showed that glenoid morphology types B2 and C predispose patients to worse radiographic results.^23^ Although our results could not confirm these findings, it must be noted that B2 glenoid was represented only in 6 (9%) cases of the total cohort and no case had type C glenoid morphology. This discrepancy may be related to differences in the FU and selection criteria regarding the extent of glenoid retroversion and humeral head subluxation.

This study had some limitations with regard to radiological implant assessment. First, the evaluation of radiological bony changes and occurrence of RLLs around the implant was only performed with conventional radiographs. There is some evidence that the radiological technique can affect the appearance of radiographs, particularly with respect to RLLs in shoulder arthroplasty.^29^^,^^47^ Considering these methodological aspects relating to the imaging processing, all radiographs were obtained preoperatively and postoperatively according to a standardized internal protocol and were performed by a board certified radiologists and technical assistants. Second, a postoperative computer tomography scan would have strengthened the precision in the detection of RLLs and periprosthetic bone adaptations.^49^ However, regarding the requirements of radiation safety and patient protection, this was not applicable in the daily clinical practice as well as in the FU period. According to a recent cadaveric study, X-ray evaluation of uncemented glenoid component is a reliable method to detect RLLs, but computer tomography scans can deliver greater sensitivity if bony changes occur.^8^ Third, a methodological limitation of this study lies in the assessment of the outcome parameters. The clinical and radiologic evaluations were performed by the authors. Therefore, the potential risk of observational bias related to the presented results could not be excluded by this study design. To obtain a more critical and unbiased assessment, further data collection in this ongoing study includes independent evaluators for radiographic analysis. Moreover, it must be mentioned that the sample size was relatively small for a multicenter study. Nevertheless, it achieved the adequate statistical power to collect meaningful results. Larger as well as midterm and long-term studies should validate these findings and expand the available radiographic and clinical data on the investigated glenoid component. Finally, the study received partial funding from Mathys Ltd. Bettlach/Enovis, Switzerland. Funds sponsored statistical analysis through an independent consultant, medical advisor contracts, and travel expenses for some of the authors. Although the company was not involved in the design or execution of the study, the analysis or interpretation of the data, or the decision to submit the results, a potential bias could exist.

## Conclusion

The use of the uncemented isoelastic monoblock Affinis Glenoid vitamys in stemless aTSA demonstrated good and reliable results, excellent clinical outcomes, and a low complication rate in a short-term FU. In addition, the radiological analysis showed a low number of RLLs around the glenoid implant. This may suggest that the biomechanical properties and design of this glenoid component could provide sufficient implant stability and survivorship over a midterm and long-term FU; however, this must be confirmed in further studies.
